# Supplementary material for: Detecting Variants in the NBN Gene While Testing for Hereditary Breast Cancer: What to Do Next?
Source: Int J Mol Sci. 2021 May 29;22(11):5832. doi: 10.3390/ijms22115832 (PMC8198239; doi:10.3390/ijms22115832)
Supplement: Supplementary file 1 [file ijms-22-05832-s001.zip › Supplementary Figure 1.pdf]

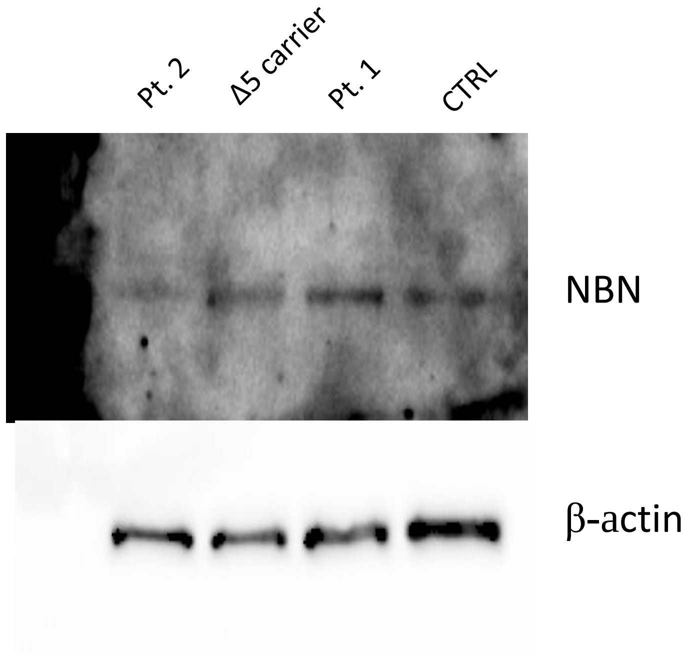

Supplementary Figure 2
Western Blot Analysis on B-LCLs of the patient 1, patient 2, a c.657del5 heterozygote carrier (Δ5 carrier) and wild-type individual (CTRL)
