# Supplementary material for: Detecting Variants in the NBN Gene While Testing for Hereditary Breast Cancer: What to Do Next?
Source: Int J Mol Sci. 2021 May 29;22(11):5832. doi: 10.3390/ijms22115832 (PMC8198239; doi:10.3390/ijms22115832)
Supplement: Supplementary file 1 [file ijms-22-05832-s001.zip › Supplementary Table 1.pdf]

Supplementary Table 1

SNP detected in the patients under study: the frequency in our sample was compared to the public population databases of Exome Aggregation Consortium (ExAC) and 1000genomes

SNP (rs dbSNP)	Chromosome position (GRCh38.p12)	Protein change	Position	Heterozigote N°	Homozigote N°	MAF in our cases	MAF in European population[1]	p-value[3]	MAF in European population[2]	p-value[3]
c.102G/A (rs1063045)	8:89982791	p.Leu34Leu	Exon 2	40	8	0.2414	0.3283	0.0060	0.3062	0.0611
c.480+24T/C (rs1805842)	8:89980710	-	Intron 4	1	0	0.0043	0	<0.0001	0	0.4229
c.553G/C (rs1805794)	8:89978251	p.Glu185Gln	Exon 5	34	9	0.2241	0.3275	0.0010	0.3062	0.0164
c.702+16G/A (rs2272581)	8:89971157	-	Intron 6	0	1	0.0086	1.584^{-05}	0.0001	0	0.0413
c.702+149T/C (rs3026271)	8:89971024	-	Intron 6	11	0	0.0474	/	/	0.0765	0.1572
c.703-18G/A (rs769418)	8:89970575	-	Intron 6	2	0	0.0086	0.03082	0.0776	0.0278	0.1392
c.896+27A/C (rs114182293)	8:89970337	-	Intron 7	1	0	0.0043	0.000308	0.1192	0.0010	0.6421
c.1124+18C/T (rs2234744)	8:89958707	-	Intron 9	39	12	0.2716	0.3277	0.0802	0.3072	0.3237
c.1124+91C/A (rs1805818)	8:89958634	-	Intron 9	39	12	0.2716	/	/	0.3062	0.3237
c.1125-79C/A (rs1805786)	8:89955634	-	Intron 9	39	9	0.2457	/	/	0.3062	0.0768
c.1197T/C (rs709816)	8:89955483	p.Asp399=	Exon 10	44	6	0.2414	0.3671	<0.0001	0.3410	0.0044
c.1915-7A/G (rs2308962)	8:89946302	-	Intron 12	38	11	0.2586	0.3284	0.0285	0.3062	0.1775
c.2016A>G (rs1061302)	8:89946194	p.Pro672=	Exon 13	36	9	0.2328	0.3280	0.0026	0.3062	0.0327

[1] Data from ExAC: http://exac.broadinstitute.org/;[2]Data from 1000genomes: http://www.1000genomes.org/category/frequently-asked-questions/population;
[3]chi-square test with Yate's correction
