# Supplementary material for: Detecting Variants in the NBN Gene While Testing for Hereditary Breast Cancer: What to Do Next?
Source: Int J Mol Sci. 2021 May 29;22(11):5832. doi: 10.3390/ijms22115832 (PMC8198239; doi:10.3390/ijms22115832)
Supplement: Supplementary file 1 [file ijms-22-05832-s001.zip › Supplementary table 2.pdf]

Hap	Allelic content	EAS	SAS	EUR	AFR	PZ	TOT
**1**	TTAGGT	32.14	43.97	65.81	13.99	54.74	38.01
**22**	CCGTAT	40.97	39.78	30.62	18.31	8.62	30.18
**7**	TTGGGT	18.06	7.46	3.48	49.77	1.72	20.94
**9**	TTGTAT	5.65	4.81	0.00	2.80	3.02	3.26
**13**	TCGTGT	0.00	0.10	0.00	5.90	0.00	1.74
**16**	CCAGGT	1.39	2.45	0.00	0.53	4.31	1.21
**19**	CCGGGT	1.59	0.41	0.00	3.33	0.43	1.43
**12**	TCGGGT	0.10	0.20	0.00	2.50	0.43	0.81
**8**	TTGTGT	0.00	0.00	0.00	1.06	6.03	0.62
**21**	CCGTGT	0.10	0.00	0.00	1.21	1.29	0.44
**17**	CCAGAT	0.00	0.00	0.00	0.00	6.47	0.33
**14**	TCGTAT	0.00	0.51	0.00	0.45	1.29	0.31
**3**	TTAGAT	0.00	0.00	0.10	0.00	4.31	0.24
**10**	TCAGGT	0.00	0.10	0.00	0.15	0.86	0.11
**5**	TTATGT	0.00	0.00	0.00	0.00	1.72	0.09
**11**	TCAGAT	0.00	0.10	0.00	0.00	0.86	0.07
**15**	CTGTAT	0.00	0.10	0.00	0.00	0.86	0.07
**6**	TTATAT	0.00	0.00	0.00	0.00	0.86	0.04
**18**	CCATGT	0.00	0.00	0.00	0.00	0.86	0.04
**2**	TTAGGC	0.00	0.00	0.00	0.00	0.43	0.02
**4**	TTAGAC	0.00	0.00	0.00	0.00	0.43	0.02
**20**	CCGGAT	0.00	0.00	0.00	0.00	0.43	0.02

EAS	SAS	EUR	AFR	PZ	TOT
324	430	662	185	127	1728
413	389	308	242	20	1372
182	73	35	658	4	952
57	47	0	37	7	148
0	1	0	78	0	79
14	24	0	7	10	55
16	4	0	44	1	65
1	2	0	33	1	37
0	0	0	14	14	28
1	0	0	16	3	20
0	0	0	0	15	15
0	5	0	6	3	14
0	0	1	0	10	11
0	1	0	2	2	5
0	0	0	0	4	4
0	1	0	0	2	3
0	1	0	0	2	3
0	0	0	0	2	2
0	0	0	0	2	2
0	0	0	0	1	1
0	0	0	0	1	1
0	0	0	0	1	1
1008	978	1006	1322	232	4546

Hap	Allelic content	EAS	SAS	EUR	AFR	PZ	TOT
**1**	TTAGGT	32.14	43.97	65.81	13.99	54.74	38.01
**2**	TTAGGC	0.00	0.00	0.00	0.00	0.43	0.02
**3**	TTAGAT	0.00	0.00	0.10	0.00	4.31	0.24
**4**	TTAGAC	0.00	0.00	0.00	0.00	0.43	0.02
**5**	TTATGT	0.00	0.00	0.00	0.00	1.72	0.09
**6**	TTATAT	0.00	0.00	0.00	0.00	0.86	0.04
**7**	TTGGGT	18.06	7.46	3.48	49.77	1.72	20.94
**8**	TTGTGT	0.00	0.00	0.00	1.06	6.03	0.62
**9**	TTGTAT	5.65	4.81	0.00	2.80	3.02	3.26
**10**	TCAGGT	0.00	0.10	0.00	0.15	0.86	0.11
**11**	TCAGAT	0.00	0.10	0.00	0.00	0.86	0.07
**12**	TCGGGT	0.10	0.20	0.00	2.50	0.43	0.81
**13**	TCGTGT	0.00	0.10	0.00	5.90	0.00	1.74
**14**	TCGTAT	0.00	0.51	0.00	0.45	1.29	0.31
**15**	CTGTAT	0.00	0.10	0.00	0.00	0.86	0.07
**16**	CCAGGT	1.39	2.45	0.00	0.53	4.31	1.21
**17**	CCAGAT	0.00	0.00	0.00	0.00	6.47	0.33
**18**	CCATGT	0.00	0.00	0.00	0.00	0.86	0.04
**19**	CCGGGT	1.59	0.41	0.00	3.33	0.43	1.43
**20**	CCGGAT	0.00	0.00	0.00	0.00	0.43	0.02
**21**	CCGTGT	0.10	0.00	0.00	1.21	1.29	0.44
**22**	CCGTAT	40.97	39.78	30.62	18.31	8.62	30.18
