# Supplementary material for: Detecting Variants in the NBN Gene While Testing for Hereditary Breast Cancer: What to Do Next?
Source: Int J Mol Sci. 2021 May 29;22(11):5832. doi: 10.3390/ijms22115832 (PMC8198239; doi:10.3390/ijms22115832)
Supplement: Supplementary file 1 [file ijms-22-05832-s001.zip › Supplementary Table 3.pdf]

Supplementary Table 3

List of cancer-predisposing genes filtered in WES analysis

Gene name	Reference sequence
APC	NM_000038
ATM	NM_000051
BARD1	NM_000465
BMPR1A	NM_004329
BRIP1	NM_032043
CDH1	NM_004360
CHEK2	NM_001005735
EPCAM	NM_002354
MLH1	NM_000249
MRE11	NM_005591
MSH2	NM_000251
MSH6	NM_000179
MUTYH	NM_012222
NBN	NM_002485
PALB2	NM_024675
PTEN	NM_000314
RAD50	NM_005732
RAD51B	NM_133509
RAD51C	NM_058216
RAD51D	NM_002878
SMAD4	NM_005359
STK11	NM_000455
TP53	NM_000546
