# Supplementary material for: Detecting Variants in the NBN Gene While Testing for Hereditary Breast Cancer: What to Do Next?
Source: Int J Mol Sci. 2021 May 29;22(11):5832. doi: 10.3390/ijms22115832 (PMC8198239; doi:10.3390/ijms22115832)
Supplement: Supplementary file 1 [file ijms-22-05832-s001.zip › Supplementary Table 4.pdf]

Name Primer	Exon	Position on hg38	
		**start**	end
NBS1-1F	1	89984756	89984737
NBS1-1R		89984386	89984367
NBS1-2F	2	89983082	89983063
NBS1-2R		89982630	89982607
NBS1-3F	3	89981631	89981616
NBS1-3R		89981236	89981217
NBS1-4F	4	89981017	89980998
NBS1-4R		89980683	89980664
NBS1-5F	5	89978421	89978400
NBS1-5R		89978152	89978132
NBS1-6F	6	89971462	89971442
NBS1-6R		89971010	89970990
NBS1-7F	7	89970664	89970642
NBS1-7R		89970299	89970275
NBS1-8F	8	89964588	89964572
NBS1-8R		89964320	89964302
NBS1-9F	9	89958964	89958946
NBS1-9R		89958611	89958593
NBS1-10F	10	89955718	89955699
NBS1-10R		89955186	89955168
NBS1-11F	11	89953771	89953748
NBS1-11R		89953198	89953174
NBS1-12F	12	89947977	89947955
NBS1-12R		89947765	89947739
NBS1-13F	13	89946370	89946350
NBS1-13R		89946080	89946057
NBS1-14F	14	89943473	89943452
NBS1-14R		89943101	89943076
NBS1-15F	15	89937194	89937175
NBS1-15R		89936920	89936900
NBS1-16F	16	89935717	89935699
NBS1-16R		89935140	89935119

Name Primer	Exon	Sequence 5'→3'
NBS1-1F_cDNA	1-2	accgatgtggaaactgctg
NBS1-2R_cDNA		caacgccagtcaaaagtctg
NBS1-10F_cDNA	10-11	tttcaccaactaaattgccaag
NBS1-11R_cDNA		tttcttcatccctttcccttt
