# Supplementary figures and images for: Detecting Variants in the NBN Gene While Testing for Hereditary Breast Cancer: What to Do Next?
Source: Int J Mol Sci. 2021 May 29;22(11):5832. doi: 10.3390/ijms22115832 (PMC8198239; doi:10.3390/ijms22115832)

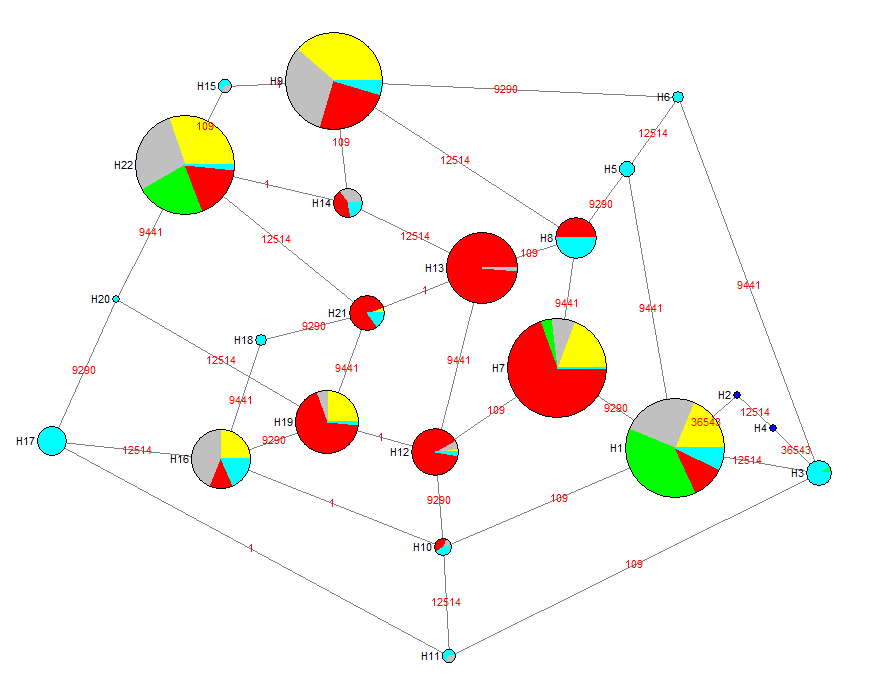

Supplement: Supplementary file 1 [file ijms-22-05832-s001.zip › Supplementray fig2.PNG]
